# EXPLORING A RANGE OF ROADMAPS LEADING TO THE AGE-FRIENDLY UNIVERSITY DESIGNATION AND BEYOND

**DOI:** 10.1093/geroni/igae098.1755

**Published:** 2024-12-31

**Authors:** Katarina Friberg-Felsted, Jacqueline Eaton, M Aaron Guest

**Affiliations:** Chair; Co-Chair; Discussant

## Abstract

The Age-Friendly University Global Secretariat encourages and processes applications to the Age-Friendly University Global Network (AFUGN). Membership indicates each institution’s commitment to endorsing the 10 Age-Friendly Principles and the implementation of age-inclusive practices to promote older adult engagement and value in the academic setting. Five presenters describe their journey in obtaining the AFU designation. They describe pre-designation road maps, steps involved, barriers faced, and implementation of post-designation plans. Dr. Orit Hirsch-Matsioulas and colleagues at the University of Haifa share how their quest aligned with the United Nations Sustainable Development Goals and reliance on deep community ties to become the first AFU institution in the Middle East. Dr. Dobish explains how mapping the 10 AFU principles to the core values of West Virginia’s Shepherd University created a successful application at a time of leadership change within the AFU Global Secretariat. Dr. Buys and colleagues delineate how Mississippi State University was invited by the State Department of Health’s Age-friendly Ecosystem Initiative to lead the pursuit of AFU designation as a sentinel university in the state. Dr. Powers illustrates how the University of Central Oklahoma’s path to AFU designation moved through both Faculty and Staff Senates and describes the university president’s investment in the inaugural post-designation initiative. Dr. Eaton and Dr. Friberg-Felsted show how garnering multi-faceted support at the University of Utah led to AFU designation and expansion into current age-inclusive collaborations. Dr. Aaron Guest, of Arizona State University and AFUGN, will facilitate discussion focused on AFU designation and implementation.

